# Alleviation of Pseudomonas aeruginosa Infection by Propeptide-Mediated Inhibition of Protease IV

**DOI:** 10.1128/Spectrum.00782-21

**Published:** 2021-10-27

**Authors:** Tae-Hyeon Kim, Xi-Hui Li, Joon-Hee Lee

**Affiliations:** a Department of Pharmacy, College of Pharmacy, Pusan National University, Busan, South Korea; Emory University School of Medicine

**Keywords:** *Pseudomonas aeruginosa*, protease IV, propeptide, infection control, animal infection model, skin infection, lung infection

## Abstract

Pseudomonas aeruginosa, an opportunistic human pathogen, expresses protease IV (PIV) for infection. Since the PIV activity can be inhibited by its propeptide, we tried to alleviate the severity of P. aeruginosa infection using the purified PIV propeptide (PIV_pp_). The PIV_pp_ treatment of P. aeruginosa could significantly inhibit the PIV activity and reduce the virulence of P. aeruginosa in multiple invertebrate infection models, such as nematodes, brine shrimp, and mealworms. The effectiveness of PIV_pp_ was further confirmed using mouse skin infection and acute/chronic lung infection models. The amount of PIV_pp_ that inhibited the PIV activity of P. aeruginosa by 65% could alleviate the severity of infection significantly in all of the skin and acute/chronic lung infections. In addition, the PIV_pp_ treatment of P. aeruginosa facilitated the healing of the skin wound infections and repressed the growth of P. aeruginosa in the infected lung. The PIV_pp_ itself did not cause the induction of inflammatory cytokines or have any harmful effects on host tissues and did not affect bacterial growth. Taken together, P. aeruginosa infections can be alleviated by PIV_pp_ treatment.

**IMPORTANCE** Pseudomonas aeruginosa is a highly antibiotic-resistant pathogen and is extremely difficult to treat. Instead of using conventional antibiotics, we attempted to alleviate P. aeruginosa infection using factors that P. aeruginosa itself produces naturally. Extracellular proteases are powerful virulence factors and important targets to control the P. aeruginosa infections. Propeptides are originally expressed as part of extracellular proteases, inhibiting their activity until they go out of the cell, preventing them from becoming toxic to the cells themselves. We confirmed, from multiple animal experiments, that treating P. aeruginosa with the purified propeptide can alleviate its infectivity. Propeptides specifically inhibit only their cognate protease without inhibiting other essential proteases of the host. The development of resistance can be avoided because the propeptide-mediated inhibition is an inherent mechanism of P. aeruginosa; hence, it will be difficult for P. aeruginosa to alter this mechanism. Since propeptides do not affect bacterial growth, there is no selective pressure to develop resistant cells.

## INTRODUCTION

Pseudomonas aeruginosa, a Gram-negative opportunistic pathogen, is found in various environments and causes various infections, such as burn wound infections, acute ulcerative keratitis, pneumonia, and many medical device-mediated infections (1, 2). Particularly, P. aeruginosa infections on burn wounds and in respiratory tracts are very common and cause severe symptoms (2, 3). In these infections, P. aeruginosa secretes many extracellular proteases as virulence factors, and among them, protease IV (PIV), elastase A (staphylolysin, LasA), and elastase B (pseudolysin, LasB) play a crucial role in pathogenesis by causing proteolytic damage to host tissues, disrupting tight junctions, and subverting host innate immunity (4, 5). Therefore, inhibiting these proteases is very important to prevent P. aeruginosa infections.

Although all three of these proteases are important, several studies have suggested that a specific inhibition of the PIV activity would alleviate *Pseudomonas* infections (4, 6, 7). PIV is a lysyl endopeptidase that cleaves the carboxyl side of lysine-containing peptides and is encoded by the *piv* gene that is highly induced by the quorum sensing system (4, 7). PIV can degrade many host proteins, including immunoglobulin, complements, fibrinogen, plasminogen, and surfactant proteins involved in the immune response to infections (4, 7, 8). As a result, PIV acts as a crucial virulence factor of P. aeruginosa in human corneal and pulmonary infections and in infections to other invertebrate hosts (4, 6, 9). Recently, PIV has been reported to exacerbate pneumococcal pneumonia and systemic disease (10).

PIV, LasA, and LasB have little similarity in amino acid sequence but have similar domain structure: a signal peptide (SP) at the N terminus, a propeptide (PP) domain in the middle, and the mature protease domain at the C terminus. They are initially expressed in full with all three domains in the cytoplasm, but their SPs and PPs are successively cleaved from the N terminus during translocation over the cytoplasmic and outer membranes to generate their mature forms (5). The full-length PIV in the cytoplasm is 48 kDa, the mature PIV is 26 kDa, and the PP of PIV (PIV_pp_) is about 19 kDa. The PPs have been suggested to specifically inhibit their own proteases (11–13). Therefore, treatment of P. aeruginosa with the purified PPs of these proteases would reduce the virulence caused by these proteases, thereby inhibiting the P. aeruginosa infection. Since LasB, PIV, and LasA are sequentially activated extracellularly, where LasB activates PIV from pro-PIV and PIV activates LasA from pro-LasA in a cascade mode (12), LasB was thought to be a better target to control P. aeruginosa infections. However, our previous *in vitro* studies have shown that the PP of LasB (LasB_pp_) was degraded by LasB itself and not able to inactivate LasB, whereas PIV and LasA were well inactivated by their own PPs (12, 13).

Therefore, in this study we tried to control P. aeruginosa infection using the purified PIV_pp_. Since PIV itself is a very strong virulence factor as well as a LasA-activating factor, it was supposedly a good target to control P. aeruginosa infection. By using various animal models, we confirmed that P. aeruginosa infection can be significantly alleviated when treated with an amount of PIV_pp_ that inhibits PIV activity by 65%.

## RESULTS

### The purified PIV_pp_ was able to inhibit the PIV activity of P. aeruginosa in live culture.

**FIG 1:**
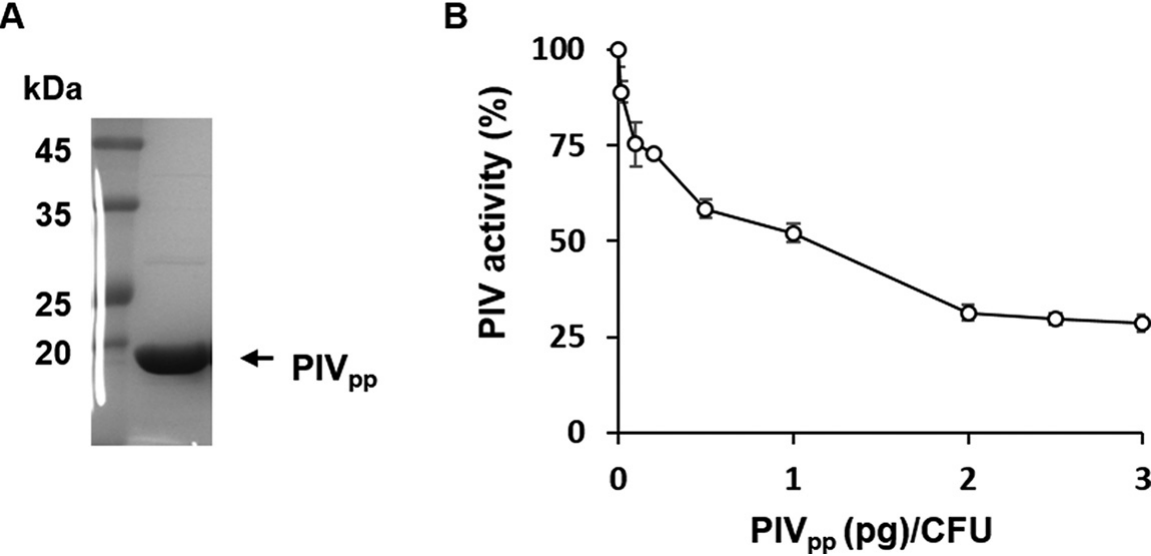
Inhibition of PIV activity in the PIV_pp_-treated P. aeruginosa. (A) The histidine-tagged PIV propeptide (PIV_pp_) was purified from E. coli BL21 and the purity of PIV_pp_ was confirmed by SDS-PAGE and densitometric analysis (>95%). (B) P. aeruginosa wild-type cells (5 × 10^5^ CFU) were treated with PIV_pp_ at the indicated ratio (pg/CFU) and the PIV activity was measured. The activity was normalized to the sample without PIV_pp_ treatment (which corresponds to 100%). Error bars mean standard deviation. This experiment was performed at least 3 times and always gave similar results.

First, we overexpressed the histidine-tagged propeptide of PIV (PIV_pp_) in Escherichia coli and purified it to a purity of >95% (Fig. 1A). In order to test whether the PIV_pp_ could inhibit the PIV activity in live P. aeruginosa culture, 5 × 10^5^ CFU of P. aeruginosa cells were treated with an increasing amount of the purified PIV_pp_. The result showed that PIV activity gradually reduced in a dose-dependent manner (Fig. 1B), indicating that PIV produced by live P. aeruginosa cells can be inhibited by the exogenous addition of PIV_pp_. At the ratio of 2 pg PIV_pp_ per CFU (1 μg of PIV_pp_), the PIV activity was inhibited by about 70% (Fig. 1B), but the growth of P. aeruginosa was not affected (data not shown).

We thought that inhibition of PIV would also reduce the activity of LasA, because PIV is a LasA-activating factor (12). In order to confirm this, we first treated 5 × 10^6^ CFU of P. aeruginosa cells with 8 μg PIV_pp_ (1.6 pg/CFU) in the exponential phase, which is the growth phase before P. aeruginosa produces proteases. Then, we further cultivated the cells for 12 h to sufficiently express proteases and measured the activities of PIV and LasA, respectively. Even under this condition, PIV activity was inhibited to less than half (Fig. S1A), and the activity of LasA was also significantly reduced (Fig. S1B). The activity of LasA was reduced to a relatively small extent, which was expected because LasB can also activate LasA in addition to PIV (12). In fact, when the LasA activity was investigated by culturing the *piv*^−^ mutant under the same conditions, the LasA activity was reduced to a level similar to that of the PIV_pp_ treatment (Fig. S1B), indicating that the PIV activity was sufficiently reduced by the PIVpp treatment under this condition.

### Treating P. aeruginosa with PIV_pp_ alleviated its virulence to small animals.

**FIG 2:**
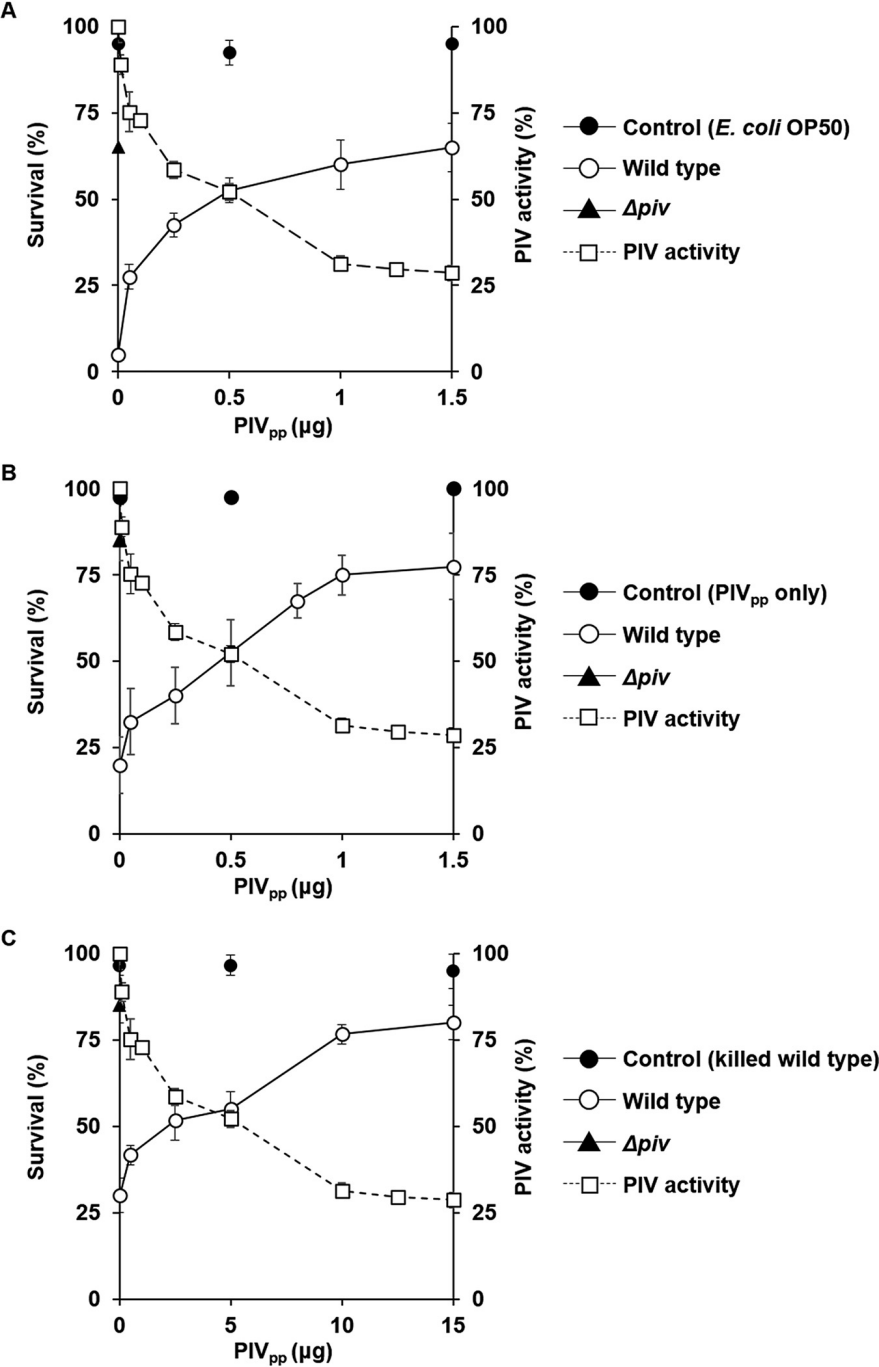
Attenuation of P. aeruginosa by PIV_pp_ treatment. P. aeruginosa wild-type cells were treated with the indicated amount of purified PIV_pp_ and used to infect C. elegans (A), *T. molitor* larvae (B), or *A. salina* nauplii (C). For infection, 5 × 10^5^ CFU of the treated P. aeruginosa cells was fed to C. elegans (A) or injected into *T. molitor* (B), or 5 × 10^6^ CFU of the treated cells was fed to *A. salina* (C). For the controls, the same CFU of E. coli OP50 (A) or heat-killed P. aeruginosa wild cells (C) were treated with PIV_pp_ and fed to C. elegans or *A. salina* in the same manner. With *T. molitor* (B), the insect saline (IS) containing 0, 0.5, and 1.5 μg of purified PIV_pp_ was injected into *T. molitor* without bacteria as the control. The same CFU of Δ*piv* strain was applied for comparison. Although the CFU and amount of PIV_pp_ were used differently in each animal experiment, the ratio was the same (these experimental conditions are summarized and compared in Table S2). C. elegans worms were transferred daily to fresh bacterial lawns and the surviving worms were counted on the 5th day (A), and the survival rates of the *T. molitor* larvae (B) and *A. salina* nauplii (C) were measured at 48 h after inoculation. The PIV activity according to the PIV_pp_ treatment in Fig. 1B was superimposed on the survival curves. Error bars mean standard deviation.

PIV has been shown to be a major virulence factor for P. aeruginosa to infect several invertebrates, such as Caenorhabditis elegans (worms, nematode), Tenebrio molitor (mealworms, insect), and Artemia salina (brine shrimp, crustacean) (4). We expected that the PIV_pp_ treatment would attenuate P. aeruginosa and increase the survival of these invertebrates in infection. When C. elegans was infected by the PIV_pp_-treated P. aeruginosa, survival dramatically increased in a dose-dependent manner (Fig. 2A). The survival rate of C. elegans in the P. aeruginosa infection was about 5% without the PIV_pp_ treatment, but it increased to about 65% with the 1.5 μg PIV_pp_ treatment (3 pg/CFU), which is a survival rate similar to that of the *piv*^−^ mutant infection (Fig. 2A). The survival rate in E. coli infection was more than 90%, and the PIV_pp_ treatment itself did not affect survival (Fig. 2A).

The infection experiments with other invertebrates, T. molitor and A. salina, showed similar results. The survival of *T. molitor* larvae with the P. aeruginosa infection was about 20% without treatment but increased to 77.5% with 1.5 μg PIV_pp_ treatment (Fig. 2B). The survival rate with the injection of insect saline containing only PIV_pp_ was always higher than 97%, indicating that PIV_pp_ itself did not have a harmful effect (Fig. 2B). The injection of the *piv*^−^ mutant also showed 85% survival (Fig. 2B). The infection experiment with brine shrimp showed a similar result. As we describe in Materials and Methods, brine shrimp were infected by adding P. aeruginosa cells into artificial seawater, and since P. aeruginosa is much diluted therein, we used CFU 10 times higher (5 × 10^6^ CFU) and treatment time two times longer (30 min) than those used in the C. elegans or *T. molitor* experiments. The survival in the P. aeruginosa infection was about 30% without treatment but increased to 80% with the treatment of 15 μg PIV_pp_ (3 pg/CFU) (Fig. 2C). The heat-killed P. aeruginosa did not kill the brine shrimp regardless of PIV_pp_ treatment (higher than 95% survival), and the *piv*^−^ mutant also showed very week virulence (85% survival) (Fig. 2C). All of these results clearly demonstrated that the treatment of P. aeruginosa with the purified PIV_pp_ can alleviate the virulence of P. aeruginosa and reduce its infectivity dose-dependently.

### PIV_pp_ alleviated the mouse skin infection by P. aeruginosa.

**FIG 3:**
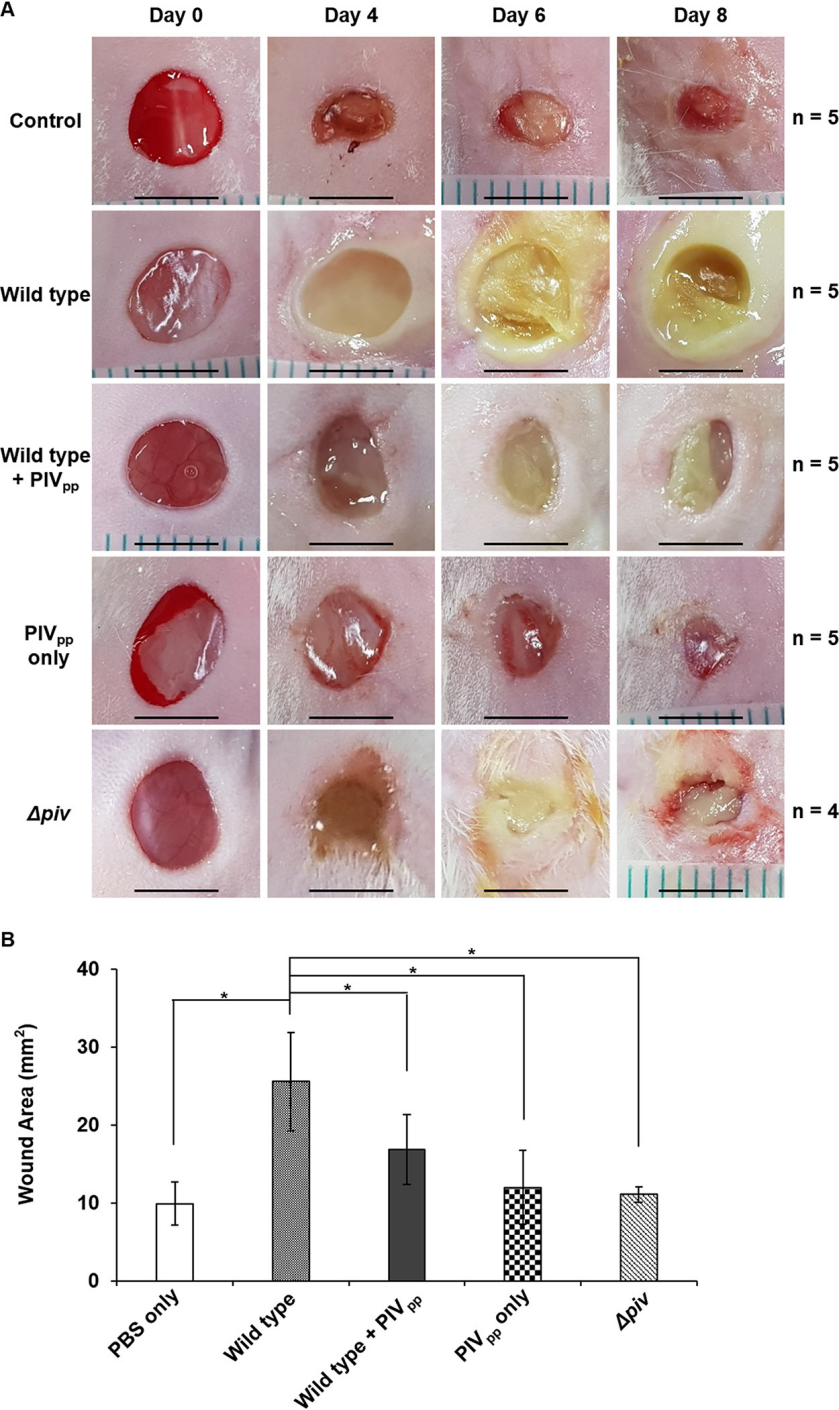
P. aeruginosa skin infection with PIV_pp_-treatment. P. aeruginosa wild-type cells (2 × 10^6^ CFU) were treated with 3.2 μg of purified PIV_pp_ (1.6 pg/CFU) and inoculated on wound sites on the skin of the mice. As controls, PBS (control), *piv*^−^ mutant cells (Δ*piv*), or PIV_pp_ alone were inoculated in the same manner. (A) Each wound site was photographed at time intervals of 0, 4, 6, and 8 days. The number of mice used in each infection experiment is indicated (*n*). Scale bar is 5 mm. (B) The wound areas were quantified from photographs using ImageJ Software. The wound areas at day 6 after infection were graphed for comparison. *, *P* < 0.05. Error bars mean standard deviation.

We used the mouse infection model to confirm the efficacy of PIV_pp_. In mouse experiments, a fixed amount of PIV_pp_ was used at 1.6 pg/CFU (0.8 μg/5 × 10^5^ CFU) for treating P. aeruginosa. This amount of PIV_pp_ reduced the PIV activity in live P. aeruginosa cells by approximately 65% and significantly increased the survival rates of small invertebrates (Fig. 2). The mouse infection studies began with skin infections, because the damages on the skin barrier by wounds often cause serious P. aeruginosa infections (14). P. aeruginosa was inoculated on 6-mm-diameter circular wound sites on the back skin of the mice, and healing was observed for several days. Without bacterial infection (control), the wound site healed in 10 days, but with the P. aeruginosa infection, the wound site was severely aggravated with pus formation (Fig. 3A). However, when mice were infected by the PIV_pp_-treated P. aeruginosa, the severity of infection was significantly reduced and the wounds were not aggravated, similar to the *piv*^−^ mutant infection (Fig. 3A and B). PIV_pp_ had no harmful effects on the wound sites when inoculated alone (Fig. 3A and B). A known strong inhibitor of PIV, TLCK (*N*-*p*-tosyl-l-chloromethyl ketone) (4, 7), was compared with PIV_pp_, but it was less effective than PIV_pp_ in alleviating P. aeruginosa infection (Fig. S2). This may be due to the toxicity of TLCK because the treatment with TLCK alone showed slower healing than the control or the PIV_pp_ alone (Fig. 3; Fig. S2). Since TLCK is an irreversible inhibitor of some important host proteases, including plasminogen (plasmin), thrombin, papain, and some kinases, including protein kinase C (PKC), its toxicity has been known for a long time (15–17). Regardless, our results demonstrated that P. aeruginosa skin infection was alleviated by the PIV_pp_ treatment.

### PIV_pp_ alleviated acute lung infection by P. aeruginosa.

**FIG 4:**
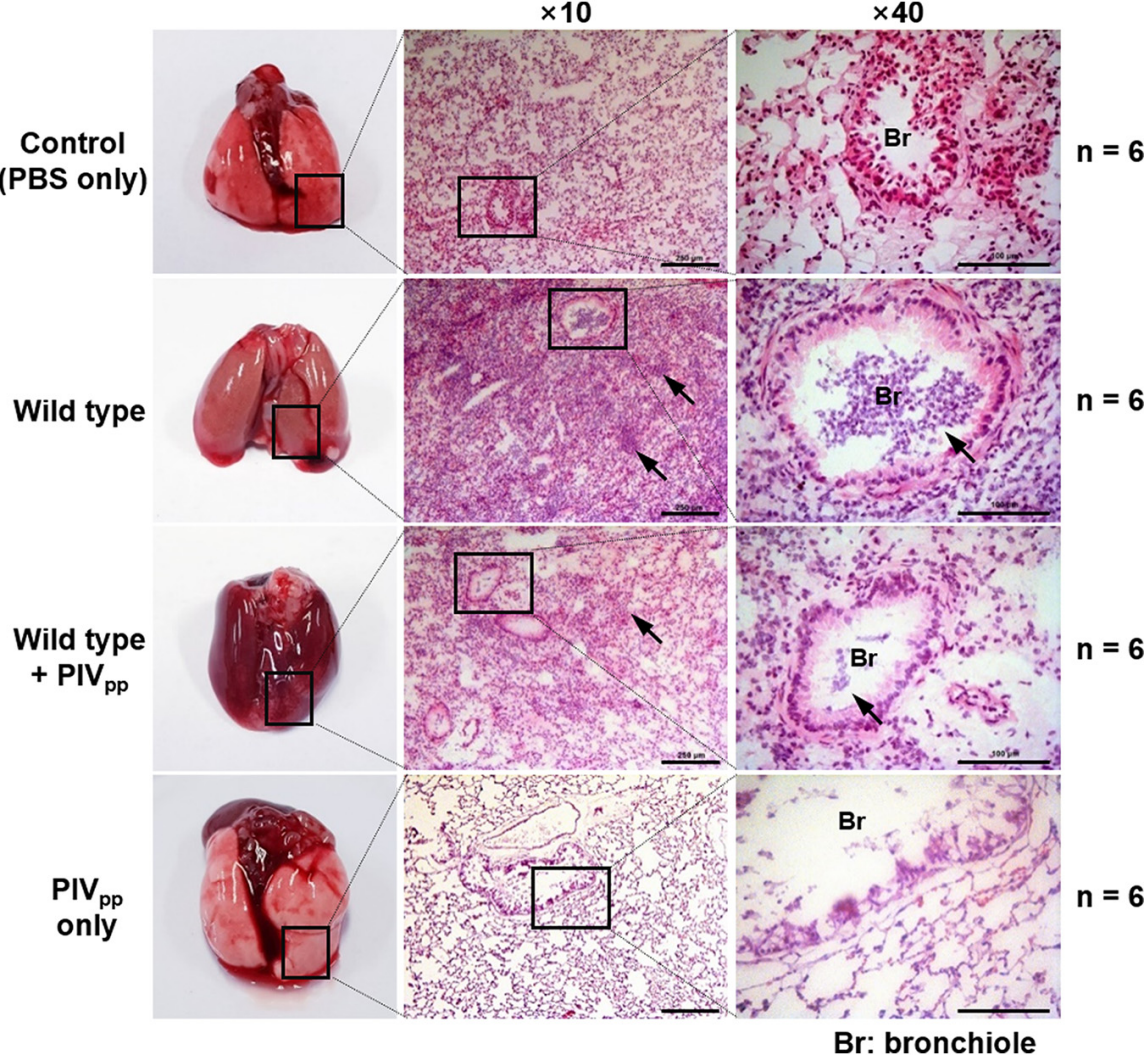
Acute lung infection with the PIV_pp_-treated P. aeruginosa. Mouse acute lung infection experiments were carried out as described in Materials and Methods. Tissues in the inferior lobes of the infected lung were stained by H&E staining and observed at two magnifications (×10 and ×40) on a microscope. Black arrows indicate the infiltrated leukocytes. The number of mice used in each infection is indicated (*n*). Scale bar is 250 μm (×10) and 100 μm (×40).

Since PIV is known to play an important role in pulmonary infections by P. aeruginosa (10), we investigated whether the PIV_pp_ treatment could alleviate the severity of infection in the mouse acute pulmonary infection model. P. aeruginosa was treated with PIV_pp_ and inoculated through the mouse trachea. Twenty-four hours after inoculation, the mice were sacrificed and their lungs were taken. The tissues were prepared from several different parts of the lungs and examined by H&E staining. The P. aeruginosa infection caused extensive inflammation in the bronchioles and pulmonary parenchyma with massive infiltration of leukocytes, whereas the bronchioles and parenchyma of the uninfected mice were clean and not inflamed (Fig. 4). When the P. aeruginosa was treated with PIV_pp_, the inflammation and infiltration of leukocytes were less severe, indicating that the PIV_pp_ treatment could alleviate the severity of infection (Fig. 4). We repeated this acute lung infection experiment independently and obtained similar results (Fig. S3 and S4). The PIV_pp_ alone did not cause inflammation or infiltration of leukocytes, indicating that the PIV_pp_ has no harmful effects (Fig. 4; Fig. S4). Like in the skin infection, the *piv*^−^ mutant caused a much less severe infection (Fig. S4). For comparison, P. aeruginosa was treated with TLCK and inoculated into the mouse trachea. While the TLCK treatment reduced the inflammation and infiltration of leukocytes to a small degree, a certain amount of inflammation and infiltration of leukocytes was observed (Fig. S4). When TLCK was administered by intraperitoneal injection instead of direct treatment, the effect of alleviating the infection was not as good as that of the direct treatment (Fig. S4).

**FIG 5:**
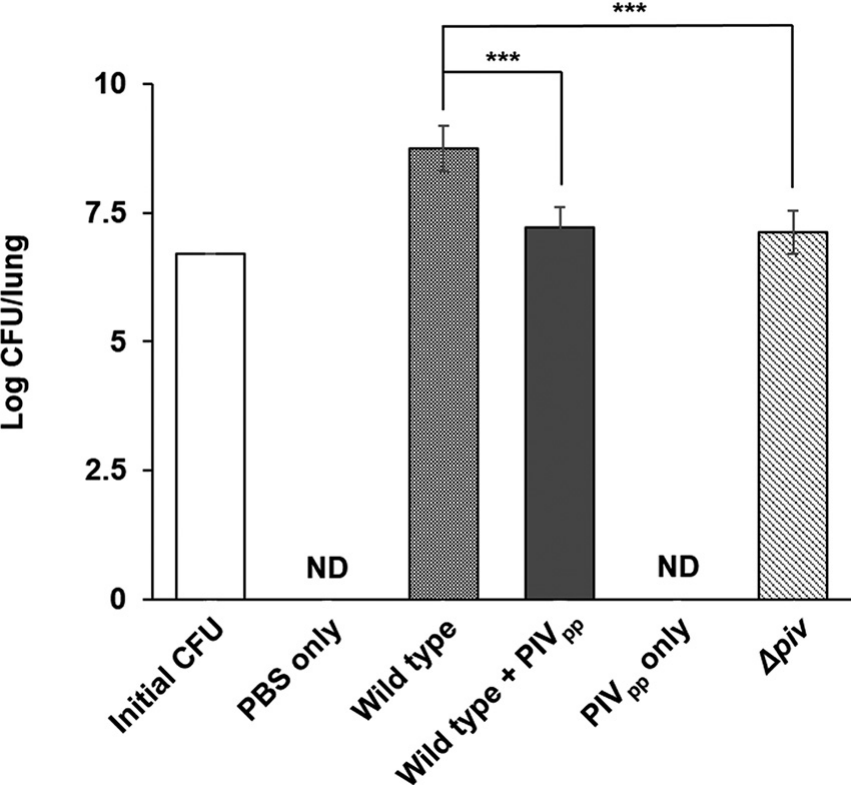
Survival of P. aeruginosa cells during the lung infections. In the acute lung infection experiments, live P. aeruginosa cells in the infected lung tissues were counted. Bacterial cells were recovered at 24 h after inoculation to each lung. The initial inoculum size (initial CFU) was 5 × 10^6^ CFU. As a control, PBS was inoculated without bacteria. The data were obtained from 4 mice for each experimental group. ***, *P* < 0.005. Error bars mean standard deviation. ND, not detected.

**FIG 6:**
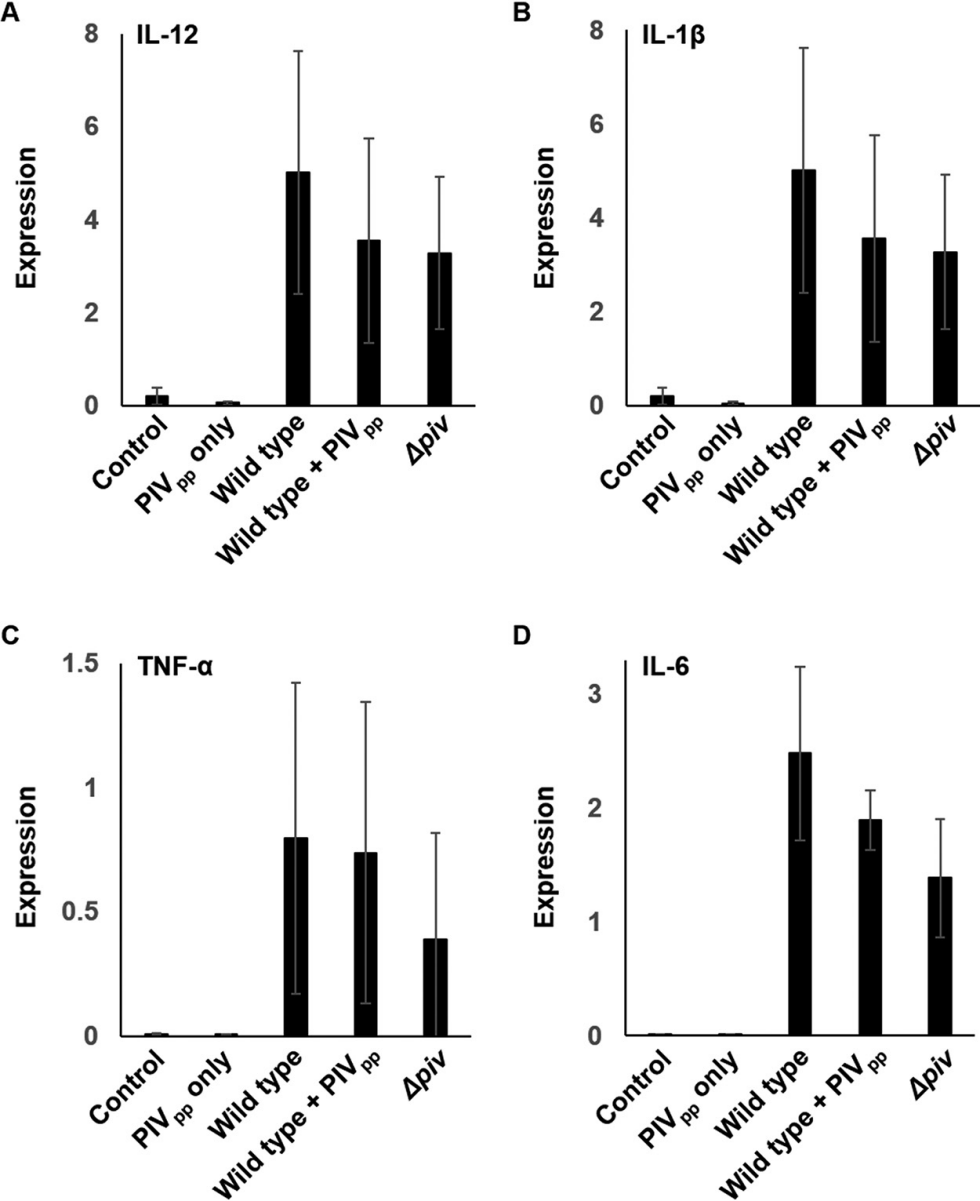
Expression of proinflammatory cytokines in the infected lungs. The mRNA levels of 4 proinflammatory cytokines, IL-12 (A), IL-1β (B), TNF-α (C), and IL-6 (D), were measured by RT-qPCR in the acute lung infection experiments and normalized by the mRNA level of β-actin. The data were obtained from 6 mice/each group. Error bars mean standard deviation.

In order to investigate the proliferation of P. aeruginosa during infection, we counted the number of live bacterial cells in the infected lungs. Compared to the number at initial inoculation, while the number of wild-type cells increased, the numbers of the *piv* mutant and PIV_pp_-treated wild-type cells did not increase (Fig. 5). This result also indicated that the PIV_pp_ treatment could repress infection. When the expressions of proinflammatory cytokines were measured, although all of the interleukin 1β (IL-1β), IL-6, IL-12, and tumor necrosis factor α (TNF-α) were greatly induced by the inoculation of P. aeruginosa, it was clear that the PIV_pp_ itself did not induce any inflammatory cytokines (Fig. 6). The difference of the PIV_pp_ treatment and the *piv* mutation was not significant, although there was a small decrease in the induction of these cytokines (Fig. 6). This is presumably because even if bacteria are attenuated, they can still induce the immune response to a similar degree.

### PIV_pp_ alleviated chronic lung infection by P. aeruginosa.

**FIG 7:**
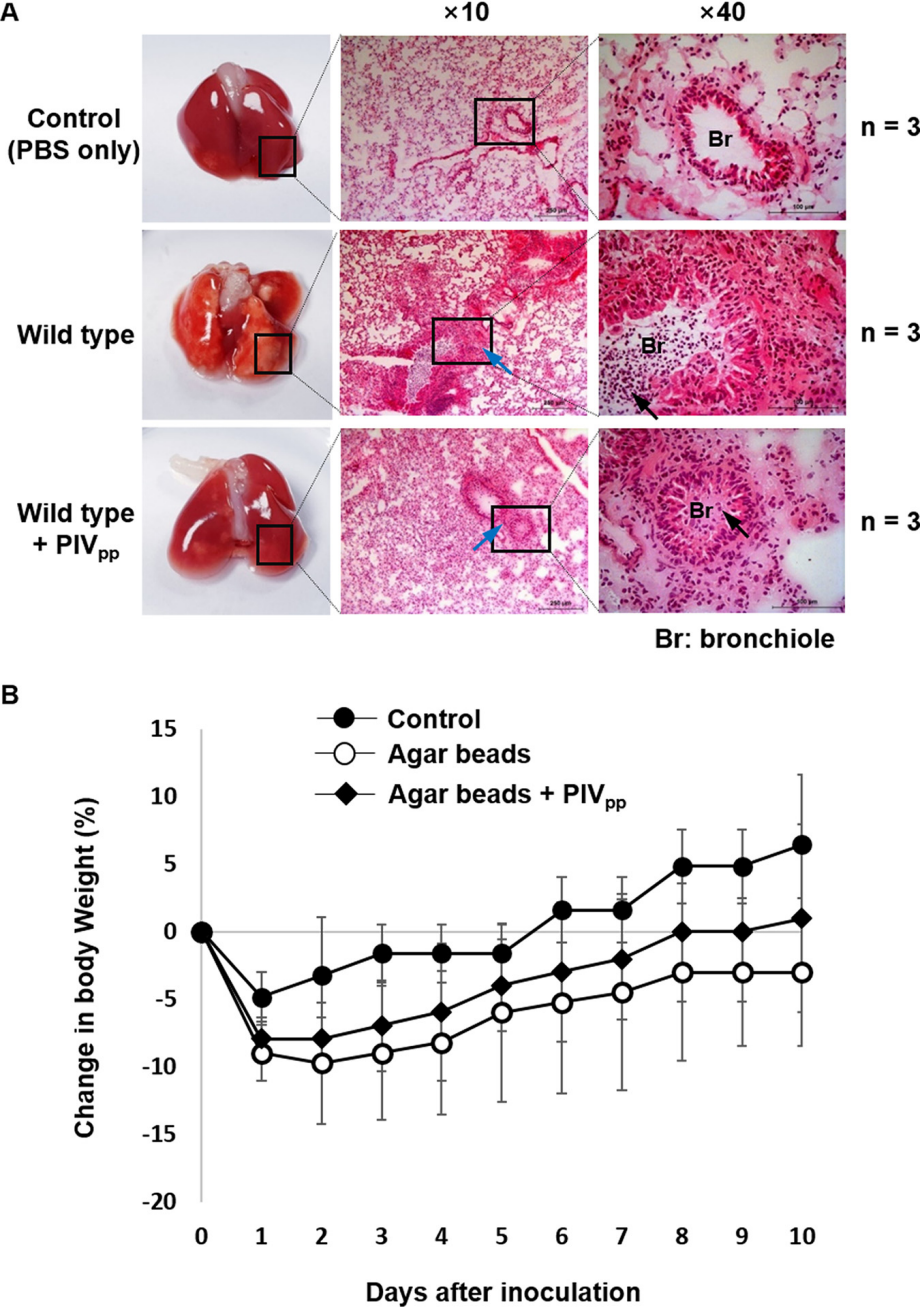
Chronic lung infection with the PIV_pp_-treated P. aeruginosa. Mouse chronic lung infection experiments were performed as described in Materials and Methods. (A) Tissues in the inferior lobes of the infected lung were prepared on the 5th day after the agar bead-mediated inoculation and stained by H&E staining. Black arrows indicate the infiltrated leukocytes and blue arrows indicate the thickened bronchiolar submucosa and alveolar walls caused by inflammation. The number of mice used in each infection is indicated (*n*). (B) The change in the body weight of the mice was measured after inoculation. Data are presented as the percent change from the initial body weight. Error bars mean standard deviation.

For a chronic lung infection experiment, P. aeruginosa cells were enmeshed in agar beads to be slowly released in the lung and then inoculated into the trachea. The result showed that the PIV_pp_ treatment alleviated the severity of the infection (Fig. 7A). However, because the experimental conditions were not as severe as those in the acute infection, the mice seemed to recover naturally without the PIV_pp_ treatment and the difference was not very distinct. The change in the body weight of the mice was also investigated. Although the recovery of body weight was slightly promoted by the PIV_pp_ treatment, the difference was small in comparison with the deviation (Fig. 7B). Although the effect of the PIV_pp_ treatment was not as great as that in the acute infection, it demonstrated that in chronic infections, the severity of the infection can still be alleviated to some extent. In conclusion, the specific inhibition of PIV by the PIV_pp_ can alleviate the severity of P. aeruginosa infections.

## DISCUSSION

This study originally aimed to relieve Pseudomonas infections by specifically inhibiting extracellular proteases. To do this, we had to make two important decisions: which protease to inhibit and how to inhibit it. PIV was a good target of choice to alleviate Pseudomonas infections for several reasons. The first is that PIV plays an important role in Pseudomonas lung and corneal infections. Second is that PIV exacerbates infections caused by other bacteria (8, 10). The third is that PIV can degrade the pulmonary surfactant proteins SP-A and SP-D. SP-A and SP-D protect the lungs by opsonizing pathogens and modulating pulmonary biophysical functions; hence, the degradation of these proteins leads to an overall decrease in lung function and can help bacteria colonize the lung (8). Additionally, an early study had suggested that specific PIV inhibitors could have a huge therapeutic value for reducing tissue damage that occurs during P. aeruginosa infections (7), therefore making PIV a good target for further research.

The inhibition method for PIV was also very important to consider. In particular, it was important to selectively inhibit only bacterial proteases, because a protease is an enzyme widely present in all living organisms. The human body also has many inherent proteases. Previous studies have demonstrated that LasB, PIV, and LasA of P. aeruginosa are specifically inhibited by their own PPs (12, 13), so the PPs were a very good tool to use for selective inhibition. PIV was inhibited not only by the PIV_pp_ naturally cleaved off from the full-length PIV but also by the exogenously added, purified PIV_pp_ (13). Therefore, in this study, the purified PIV_pp_ was tested for the therapeutic application to control P. aeruginosa infection, and the results showed that it can relieve the infection and even has a better effect than TLCK, a general serine protease inhibitor.

In addition to this specific inhibition, the use of PPs has the additional, important advantage of being able to avoid the generation of resistance. The PP-mediated inhibition of the proteases is an inherent mechanism of P. aeruginosa to prevent their premature activation inside the cell and hence to protect cells from the undesired degradation of their own proteins (12, 13). Therefore, it would be difficult for P. aeruginosa to eliminate or alter this PP-related mechanism, and thus it would rarely develop resistance to PP treatment. Moreover, since the PP treatment inhibits only virulence without killing P. aeruginosa or restricting its growth, it does not give a selective pressure to enrich the resistant bacterial cells.

As mentioned earlier, since LasB, PIV, and LasA are sequentially activated in a cascade manner, with LasB as the initial factor triggering the activation of PIV and LasA (12, 13), the activity of all three proteases could be inhibited at the same time with the inhibition of LasB. Regretfully, however, LasB was not inhibited by exogenous LasB_pp_, unlike PIV. Once activated, LasB can degrade its own LasB_pp_, so it has resistance to the inhibition by LasB_pp_ (12). Therefore, we inhibited PIV as an alternative target using the PIV_pp_ in this study.

The PIV_pp_ effect was well observed in acute lung infections but less apparent in chronic lung infections. P. aeruginosa infections can be either acute or chronic; acute infections usually involve virulence factors, including proteases, are invasive and cytotoxic, and frequently result in massive tissue destruction (1, 2, 4, 18). Bacterial virulence factors, including proteases, are primarily working in acute infections, which may account for the well-observed effect of the PIV_pp_ treatment. On the other hand, chronic P. aeruginosa infections are usually caused by resistance to host immunity and progressed by host factors rather than by various virulence factors. Chronic infections are often mediated by biofilms, which make P. aeruginosa more resistant to host immunity and antibiotic medications (18). Judging by these results, protease activity may be considered more important in acute infections.

We also want to mention that there are points that need to be improved in using PPs as therapeutic agents. Although the PP is a protein that is not large, it can be immunogenic when used repeatedly. This is a common disadvantage of all protein-based drugs. To overcome this drawback, it is necessary to find the most important core moiety required to inhibit PIV and to minimize the size of the PP because, generally, the smaller the protein, the less immunogenic.

## MATERIALS AND METHODS

### Bacterial strains, plasmids, and culture conditions.

Bacterial strains, model animals, primers, and plasmids used in this study are listed in Table S1. Bacterial cells were generally grown in Luria-Bertani medium (LB; yeast extract 0.5%, bacto-tryptone 1%, NaCl 0.5%) at 37°C with vigorous shaking. For some experiments, 3% Bacto tryptic soy broth (TSB; BD) was used. Agar was added at 1.5% (wt/vol) to solidify the media. Bacterial growth was measured by optical density at 600 nm (OD_600_). To prepare the P. aeruginosa cells for infection, cells were cultivated up to an OD_600_ of 3.0 with vigorous shaking at 37°C, harvested by centrifugation, and resuspended in sterilized phosphate-buffered saline (PBS). Ampicillin and tetracycline were added at 100 μg/ml and 50 μg/ml, respectively. IPTG (isopropyl-1-thio-β-d-galactopyranoside) was added at 0.5 mM for protein induction.

### Overexpression and purification of protease IV propeptide.

The PIV_pp_ was overexpressed in E. coli BL21(DE3) using pET16b-PIVpro (Table S1) and purified by a nickel-nitrilotriacetic acid (Ni-NTA) column (Invitrogen) as described previously (13). The fractions containing pure PIV_pp_ (Fig. 1A) were collected, dialyzed in storage buffer (20 mM Tris-HCl [pH 8.0]), and stored at −80°C.

### PIV activity assay.

PIV activity was measured using a chromogenic substrate [plasmin, *N*-(*p*-tosyl)-Gly-Pro-Lys-4-nitroanilide acetate salt; Sigma-Aldrich] as described previously (13). When cleaved by PIV, this substrate releases nitroanilide that can be measured by absorbance at 410 nm (*A*_410_). P. aeruginosa PAO1 cells (5 × 10^5^ CFU) and various amounts of purified PIV_pp_ were mixed in 100 μl of 50 mM Tris-HCl (pH 8.0) containing 200 μM chromogenic substrate and incubated at 37°C for 30 min. The viability or growth of P. aeruginosa was unaffected during the chromogenic assay. The PIV activity was measured by *A*_410_ using a spectrophotometer (Optizen POP, Mecasys, Daejeon, South Korea). For a more intuitive understanding, the PIV activity was relatively presented as a percentage (100%, the activity of wild type without treatment; 0%, the activity of *piv*^−^ mutant).

### LasA activity assay.

The LasA activity was determined by a staphylolysis assay (12). The overnight cultured Staphylococcus aureus cells were resuspended in 25 mM diethanolamine (pH 9.5) and heat-killed at 100°C for 10 min. The culture supernatants of PIV_pp_-treated P. aeruginosa were mixed with the heat-killed S. aureus cells (OD_600_ = 1.0). After 1.5-h incubation at 37°C, the extent of S. aureus cell lysis was determined by measuring OD_600_. Since the LasA activity is higher at lower OD_600_ values in this method, the activity of LasA was converted to the relative value of LasA activity in the untreated wild type using the following equation: LasA activity (%) = [(1 − OD_600_ of reaction with sample)/(1 − OD_600_ of reaction with untreated wild type)] × 100.

### Virulence assay with Caenorhabditis elegans.

C. elegans worms were routinely grown on bacterial lawns of E. coli OP50 strain on NGM (nematode growth medium) agar plates (1 mM CaCl_2_, 1 mM MgSO_4_, 3 g/liter NaCl, 17 g/liter, 2.5 g/liter peptone, 17 g/liter agar, and 5 mg/liter cholesterol) at 20°C. For virulence analysis, 5 × 10^5^ CFU of P. aeruginosa cells was mixed with 0, 0.05, 0.25, 0.5, 1, and 1.5 μg of purified PIV_pp_ at the ratio of 0, 0.1, 0.5, 1, 2, and 3 pg/CFU, respectively. As a control, the same amount of CFU of E. coli OP50 was mixed with purified PIV_pp_ at the ratio of 0, 1, and 3 pg/CFU. After 15-minute incubation at room temperature, whole cells were spread on NGM agar plates to form bacterial lawns. The lawn formation was unaffected by PIV_pp_. As another control, the same amount of CFU of the *piv* mutant (Δ*piv*) was spread on the NGM agar plates without the PIV_pp_ treatment. Forty C. elegans worms at larval stage 4 (L4) were placed on each bacterial lawn and incubated at 20°C with daily transfer to fresh lawns, and alive/dead worms were counted daily. The PIV_pp_ did not affect the bacterial growth (data not shown).

### Virulence assay with *Tenebrio molitor*.

*T. molitor* larvae were grown on wheat bran at 25°C. For virulence assay, P. aeruginosa cells (5 × 10^5^ CFU) that were treated with PIV_pp_ in the same manner as that described above were carefully injected into *T. molitor* larvae using a syringe. As a control, the same volume of insect saline (1 mM CaCl_2_, 130 mM NaCl, 5 mM KCl) containing 0, 0.5, and 1.5 μg of purified PIV_pp_ was injected. The larvae were further incubated in petri dishes at 25°C and the live/dead were counted for several days. Forty larvae were used for each experimental group.

### Virulence assay with brine shrimp (*Artemia salina*).

*A. salina* specimens were purchased as dormant eggs or cysts (Artemio mix, JBL, Germany). About 3.2 g of the cysts premixed with sea salts was suspended in 166 ml of sterilized water and incubated at 25°C for 24 h. The eggs then hatched and grew to nauplii. The nauplii were further cultivated in artificial seawater prepared by dissolving 35 g of sea salts (Sigma-Aldrich, S9883) in 1 liter of sterilized water. P. aeruginosa virulence was measured using nauplii as described previously, with minor modifications (19). The nauplii were transferred into 5 ml of artificial seawater in a petri dish (35 by 10 mm; 20 nauplii per dish). These nauplii were infected by adding P. aeruginosa cells into seawater, but since P. aeruginosa is much diluted therein, we used more cells (5 × 10^6^ CFU) and longer treatment time (30 min) than in the C. elegans or *T. molitor* experiments. A total of 5 × 10^6^ CFU of P. aeruginosa cells was treated with 0, 0.5, 2.5, 5, 10, and 15 μg of purified PIV_pp_ at the same ratio (0, 0.1, 0.5, 1, 2, and 3 pg/CFU) at 25°C for 30 min and added into artificial seawater containing nauplii at 5 × 10^6^ CFU/ml for infection. As a control, heat-inactivated wild-type cells treated with 0, 5, and 15 μg of purified PIV_pp_ (0, 1, and 3 pg/CFU) or *piv*^−^ mutant cells were added at 5 × 10^6^ CFU/ml. While incubating at 25°C, the survival of nauplii were counted daily for several days. Sixty nauplii were used for each virulence assay.

### Mouse skin infection experiment.

Male Jcl:ICR mice (Samtako Bio, South Korea; Table S1) were maintained in polycarbonate cages (4 to 5 mice per cage) with wood chip bedding at 24°C and 55% humidity in a 12-h light/dark cycle. Seven- to nine-week-old mice were randomly divided into 7 groups (1 to 5 mice per each group) and assigned for the inoculation of PBS (bacteria-free control), P. aeruginosa wild type, *piv*^−^ mutant, PIV_pp_-treated wild type, PIV_pp_ alone, TLCK-treated P. aeruginosa, and TLCK alone, respectively. The mice were anesthetized by 1.2% avertin (2,2,2-tribromoethanol) solution for 20 min. Dosage of avertin was 0.2 ml/10 g body weight (20). The hair on the back skin was shaved and disinfected with 70% ethanol. Then, circular wounds (6 mm diameter) were made via biopsy punch (Kai Medical, Japan) (21), and 2 × 10^6^ CFU of P. aeruginosa cells or other controls in 20 μl was dropped onto the wound sites. For the treatment of PIV_pp_ or TLCK, 2 × 10^6^ CFU of P. aeruginosa cells was mixed with 3.2 μg of PIV_pp_ (1.6 pg/CFU) or 1 mM TLCK in 20 μl at room temperature for 15 min before inoculation of the wound sites. The wound sites were observed and photographed for 10 days at 2-day intervals. The area of each wound was quantified from the photographs using ImageJ Software (National Institutes of Health, Bethesda, MD, USA).

### Mouse acute lung infection experiment.

Six-week-old male Jcl:ICR mice were randomly divided into each experimental group (1 to 6 mice per each group) and anesthetized as described above. Each group was inoculated by PBS (control), P. aeruginosa, *piv*^−^ mutant, PIV_pp_-treated P. aeruginosa, PIV_pp_ alone, TLCK-treated P. aeruginosa, TLCK alone, P. aeruginosa with TLCK intraperitoneal (i.p.) injection, and TLCK i.p. injection alone, respectively. The inoculum size was 5 × 10^6^ CFU, which was mixed with 8 μg of purified PIV_pp_ (1.6 pg/CFU) or 1 mM TLCK in 50 μl PBS and incubated at room temperature for 15 min. As a control, 50 μl of PBS containing 1 mM TLCK was inoculated without bacterial cells. The i.p. injection of TLCK was done via syringe at 10 mg/weight (kg) in 200 μl independent of the tracheal inoculation of 5 × 10^6^ CFU cells. For intratracheal inoculation, the mice were placed in supine position and their necks were disinfected with 70% ethanol. The neck was cut vertically and the trachea was opened, into which samples (in 50 μl solution) were inoculated via syringe. The incision was closed using Skin Stapler (Visistat, USA) and the mice were placed on a heating pad at 37°C until awake (22). At 24 h after inoculation, the infected mice were sacrificed for lung harvest.

### Mouse chronic lung infection experiment.

The mouse chronic lung infection was performed by embedding P. aeruginosa cells into agar beads for slow release before tracheal injection (22). P. aeruginosa was grown overnight, 1:50-diluted into 20 ml fresh LB medium, and cultivated up to an OD_600_ of 1.2. P. aeruginosa cells were harvested by centrifugation (6,000 × *g* at 4°C) and completely resuspended in 1 ml of sterile PBS. A total of 9 ml of TSB containing 1.5% agar was autoclaved and cooled down to 50°C in a water bath. The PBS-resuspended P. aeruginosa cells were added to this TSB agar and this mixture was mixed with 200 ml of prewarmed sterile mineral oil. This mixture was immediately stirred with a magnetic bar for 6 min at room temperature, slowly cooled down to 4°C with constant stirring (about 80 rpm), and placed on ice. Once agar beads were formed, they were transferred into 50-ml conical tubes and harvested by centrifugation (6,000 × *g*, at 4°C). After complete removal of mineral oil by 6 different washes with sterile PBS, the agar beads were resuspended in 25 ml PBS. Bacterial CFU contained in the agar beads was measured by plate colony count after the beads were homogenized with brief sonication. The agar beads were stored at 4°C until use. For inoculation in mice, the agar beads were diluted to 5 × 10^6^ CFU in 30 μl PBS and injected into the mouse trachea in the same manner as that used in the acute lung infection. For the PIV_pp_ treatment, the agar beads containing 5 × 10^6^ CFU were incubated with 8 μg of the purified PIV_pp_ (1.6 pg/CFU) in 50 μl at room temperature for 15 min. Nine mice were randomly divided into three groups (3 mice for each group) and inoculated with the agar beads containing PBS, P. aeruginosa, and the PIV_pp_-treated P. aeruginosa, respectively. After inoculation, the mice were cared for as described in the acute lung infection with body weights recorded daily and sacrificed on the 5th day after the inoculation for lung harvest.

### Lung section, microscopic observation, and bacterial count with infected lungs.

The lung section for histological examination was carried out as described elsewhere (23). After the infected mice were euthanized by CO_2_, their lungs were removed, fixed in 4% formalin, and stored at 4°C overnight. For histological observation, lobes of the lungs were cut and soaked in PBS containing sucrose with a gradual increase of sucrose concentration (10% for 1 h, 20% for 2 h, and 30% overnight at 4°C). After the solution was completely removed, the lobes were frozen in OCT compound (Tissue-Tek, USA) and sectioned into 7-μm-thick slices using a cryostat microtome (CM1860, Leica, Germany) for hematoxylin and eosin (H&E) staining, as described elsewhere (22, 23). The slices were washed with water and first stained in the Mayer’s hematoxylin solution {50 g aluminum potassium sulfate [KAl(SO_4_)_2_•12H_2_O], 1 g hematoxylin, 0.2 g sodium iodate [NaIO_3_], 1 g citric acid [monohydrate], H_2_O [to 1,000 liters]} for 8 min at room temperature. After enough washes in water and 95% ethanol, the slices were counterstained in eosin Y solution (eosin Y stock [1% eosin Y in 76% EtOH]:80% EtOH:glacial acetic acid = 50:150:1). After dehydration in absolute ethanol, the slices were soaked in xylene (DAEJUNG, South Korea) to make the stain more vivid, mounted with castor oil, covered by cover glass, and observed on a microscope (CX40, Olympus, Japan). To determine the bacterial count in the infected lungs, the lungs were removed at 24 h after intratracheal inoculation of P. aeruginosa in acute infection experiments and homogenized in l ml PBS with TissueLyser II (Qiagen, Germany) at 30 Hz for 20 s. The number of bacteria in the homogenized lungs was determined by plate colony count.

### RNA extraction and RT-qPCR analysis.

Total RNA was extracted from mouse lung tissue (50 mg) using RiboEx (GeneAll, South Korea) according to the manufacturer’s instructions. The RNA was dissolved in RNase-free water and stored at −80°C. cDNA was synthesized from 2 μg of the RNA using SuPrimeScript RT Premix (GeNet Bio, South Korea). The real-time qPCR (RT-qPCR) was carried out with the synthesized cDNA, specific primers (Table S1), and SensiFAST SYBR No-ROX kit (Bioline, UK) by using the CFX Connect real-time system (Bio-Rad Laboratories Inc., USA). The level of mRNA expression was normalized by the expression level of β-actin as an internal control.

### Ethics statement.

Mice were cared for in a laboratory animal facility at Pusan National University. All animal experiments were done in accordance with the National Institutes of Health (NIH, USA) guidelines, and the animal protocol used in this study was reviewed and approved beforehand by the Pusan National University-Institutional Animal Care Committee (PNU-IACUC) with respect to ethicality and scientific care (approval number: PNU-2018-2019).

### Statistical analysis.

In order to ensure the significance of the results, the data were statistically analyzed using Student’s *t* test (two-sample assuming equal variances) in MS Office Excel (Microsoft, USA). If the *P* value was lower than 0.05, it was considered significant.
