# Supplementary material for: Alleviation of Pseudomonas aeruginosa Infection by Propeptide-Mediated Inhibition of Protease IV
Source: Microbiol Spectr. 2021 Oct 27;9(2):e00782-21. doi: 10.1128/Spectrum.00782-21 (PMC8549743; doi:10.1128/Spectrum.00782-21)
Supplement: SUPPLEMENTAL FILE 1 — Supplemental material. Download SPECTRUM00782-21_Supp_1_seq1.pdf, PDF file, 1.5 MB [file spectrum00782-21_supp_1_seq1.pdf]

Alleviation of *Pseudomonas aeruginosa* infection by propeptide-mediated inhibition of Protease IV

Tae-Hyeon Kim, Xi-Hui Li, and Joon-Hee Lee*

Department of Pharmacy, College of Pharmacy, Pusan National University, Busan, 46241, South Korea

Supplementary material: Table S1, Table S2, Fig. S1, Fig. S2, Fig. S3, and Fig. S4

Table S1. Organisms, plasmids, and primers used in this study.

Names	Description	References
**Bacteria**		
Pseudomonas aeruginosa		
PAO1	Wild type strain of *P. aeruginosa*	(1)
DH0001	*piv*$^-$ mutant of PAO1, Tc^R	(2)
Staphylococcus aureus		
RN4220	Wild type of *S. aureus*	(3)
Escherichia coli		
DH5α	*supE44*Δ*lacU*169(80*lac*ZΔM15)*hsdR17 recA1 gyrA9 6thi-1 relA1*	Lab. collection
OP50	Uracil auxotroph *E. coli* B	(4)
BL21(DE3)	F$^-$ *omp*T *hsd*S$_B$ (r$_B^-$ m$_B^-$) *dcm gal* λ (DE3)	Lab. collection
**Small animals**		
Caenorhabditis elegans	Roundworm, N$_2$ strain	Lab. collection
Tenebrio molitor	Yellow mealworm	(2)
Artemia salina	A species of brine shrimp	Artemio® Mix, JBL, Germany
**Mouse**		
Jcl:ICR	A strain of albino mice	Samtako Bio, Korea
**Plasmid**		
pET16b-PIVpro	Propeptide-coding region of PIV in pET16b, Ap^R	(5)
**Primers (F: forward, R: reverse, 5'-3')**		
β-actin	F: TCACACACTGTCCCCATCTACG<br>R: ACCACGCTCGGTCAGGATTTTC	(6)
TNF-α	F: CATCTTCTCAAAATTCGAGTGACAA<br>R: TGGGAGTAGACAAGGTACAACCC	(7)
IL-1β	F: GCCCATCCTCTGTGACTCAT<br>R: AGGCCACAGGTATTTTGTCG	(8)
IL-6	F: CAGAATTGCCATCGTACAACTCTTTTCTCA<br>R: AAGTGCATCATCGTTGTTCATACA	(7)
IL-12p40	F: GGAAGCACGGCAGCAGAATA<br>R: AACTTGAGGGAGAAGTAGGAATGG	(7)

Tc, Tetracycline; Ap, Ampicillin

Table S2. Summary of the experiments used in this study.

	In vitro	Animal infection experiments					
Infection target	-	*C. elegans*	*T. molitor*	*A. salina*	Mouse skin	Mouse lung (acute)	Mouse lung (chronic)
Bacterial dose for infection (CFU)	5×10^6	5×10^5	5×10^5	5×10^6	2×10^6	5×10^6	5×10^6 (in agar bead)
PIV_{pp} amounts for bacterial treatment (µg)	8	0, 0.05, 0.25, 0.5, 1, 1.5	0, 0.05, 0.25, 0.5, 1, 1.5	0, 0.5, 2.5, 5, 10, 15	3.2	8	8
PIV_{pp} per CFU (pg/CFU)	1.6	0, 0.1, 0.5, 1, 2, 3	0, 0.1, 0.5, 1, 2, 3	0, 0.1, 0.5, 1, 2, 3	1.6	1.6	1.6
Non-pathogen control	-	*E.coli* OP50 with 0, 0.5, 1.5 µg of PIV_{pp}	Insect saline with 0, 0.5, 1.5 µg of PIV_{pp}	Heat-killed *P. aeruginosa* PAO1 with 0, 5, 15 µg of PIV_{pp}	PBS with or without 3.2 µg of PIV_{pp}	PBS with or without 8 µg of PIV_{pp}	PBS with or without 8 µg of PIV_{pp}
PIV-deficient control	-	*Δpiv*	*Δpiv*	*Δpiv*	*Δpiv*	*Δpiv*	-

Fig. S1

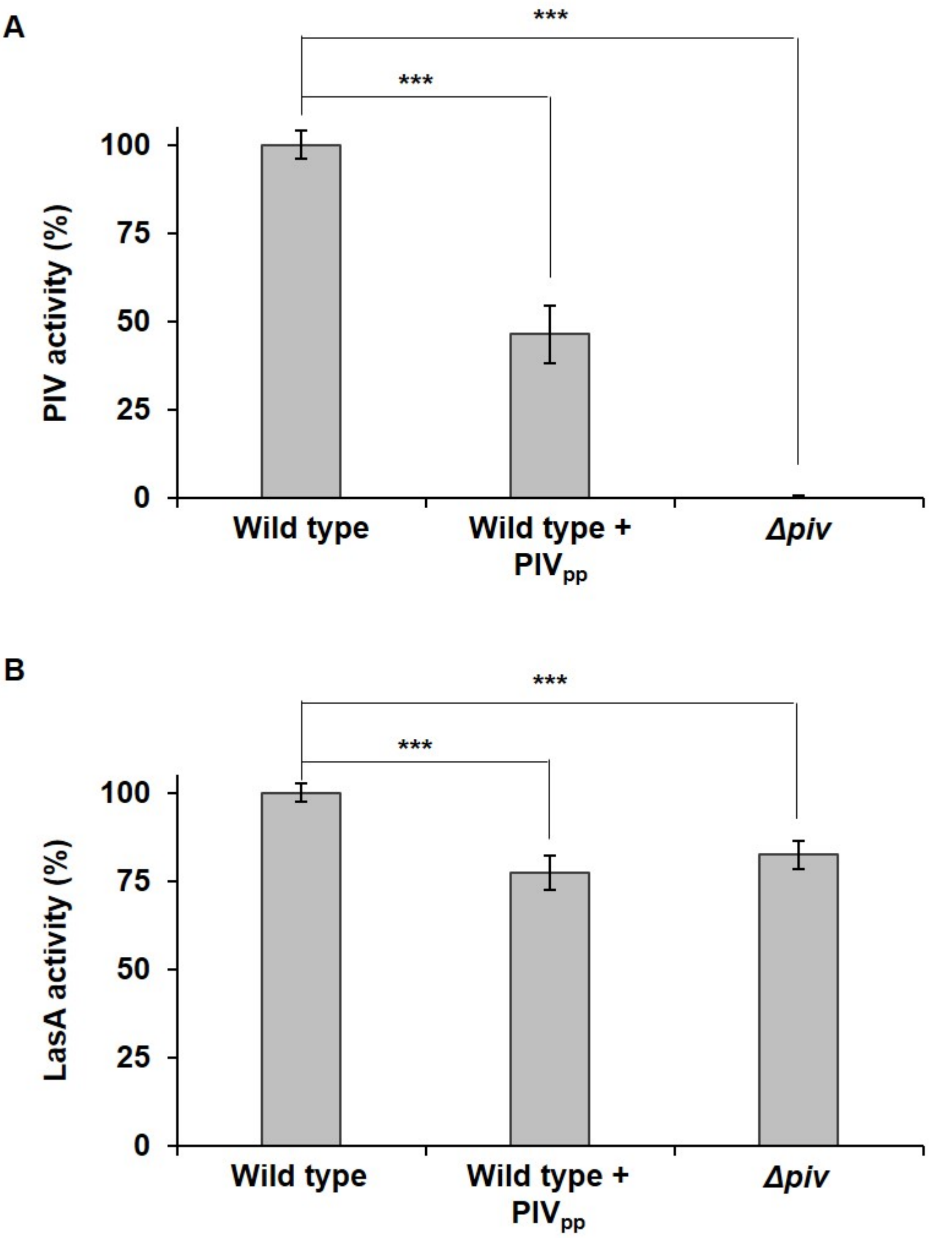

Fig. S1. Inhibition of both PIV and LasA by the PIV$_{pp}$-treatment. *P. aeruginosa* wild type and *Δpiv* mutant cells were cultured in fresh LB up to OD_{600} = 0.5. Then, 5 × 10^6 CFU of the cells were taken and mixed with 8 μg of purified PIV$_{pp}$ (1.6 pg/CFU). Cells were further incubated at 37 °C for 12 hours to enable cells to express PIV and LasA sufficiently. The activities of PIV (A) and LasA (B) were measured as described in Materials and Methods. ***, $p < 0.005$. Error bars mean standard deviation.

Fig. S2.

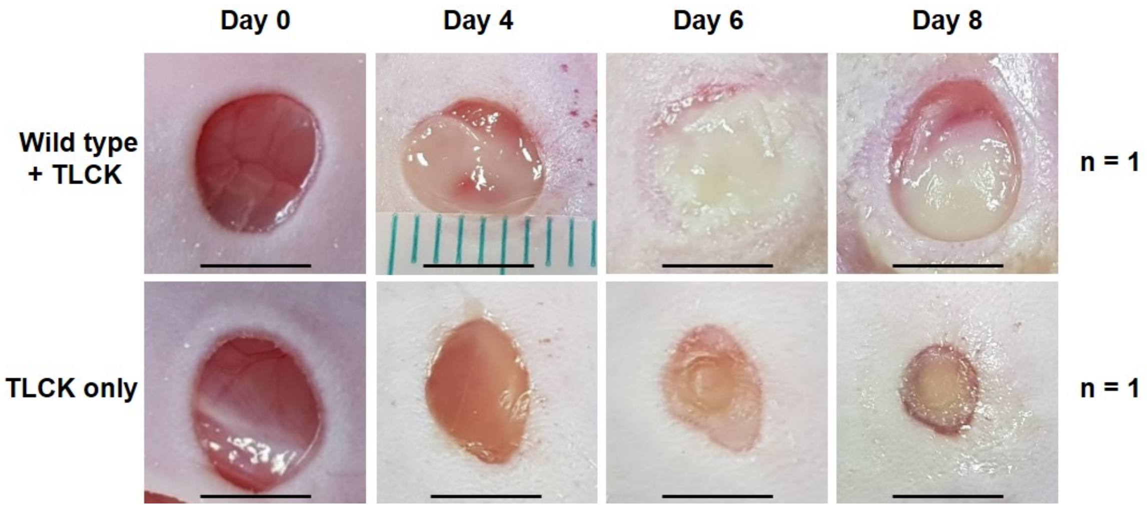

Fig. S2. Skin infection with TLCK-treated *P. aeruginosa*. *P. aeruginosa* wild type cells (2 × 10^6 CFU) were treated with 1 mM TLCK and inoculated on the circular wound sites on the skin of the mice. The same volume of 1 mM TLCK was dropped alone on the wound site as a control.

Fig. S3

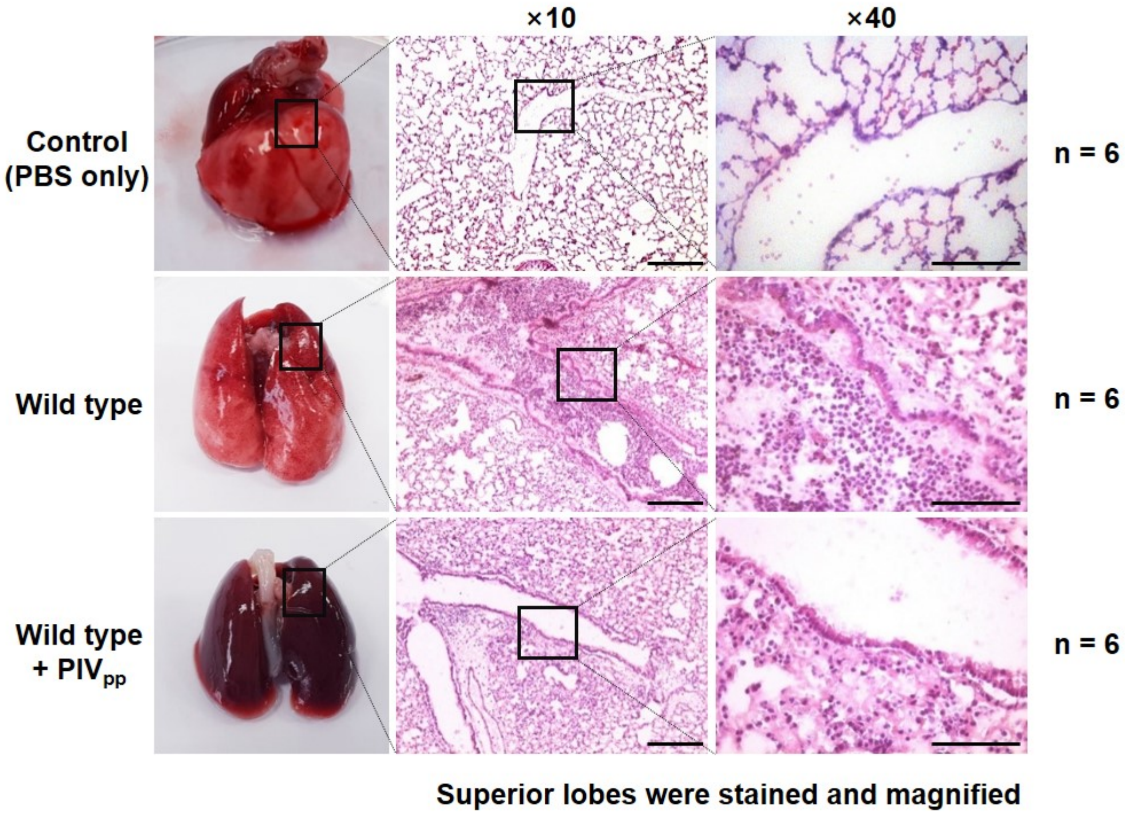

Fig. S3. 2nd trial of the *P. aeruginosa* acute lung infection experiment. The *P. aeruginosa* acute lung infection experiments in Fig. 4 were independently repeated, in which superior lobes were stained and observed in this trial, and similar results were obtained. The number of mice used in each infection were indicated (n).

Fig. S4

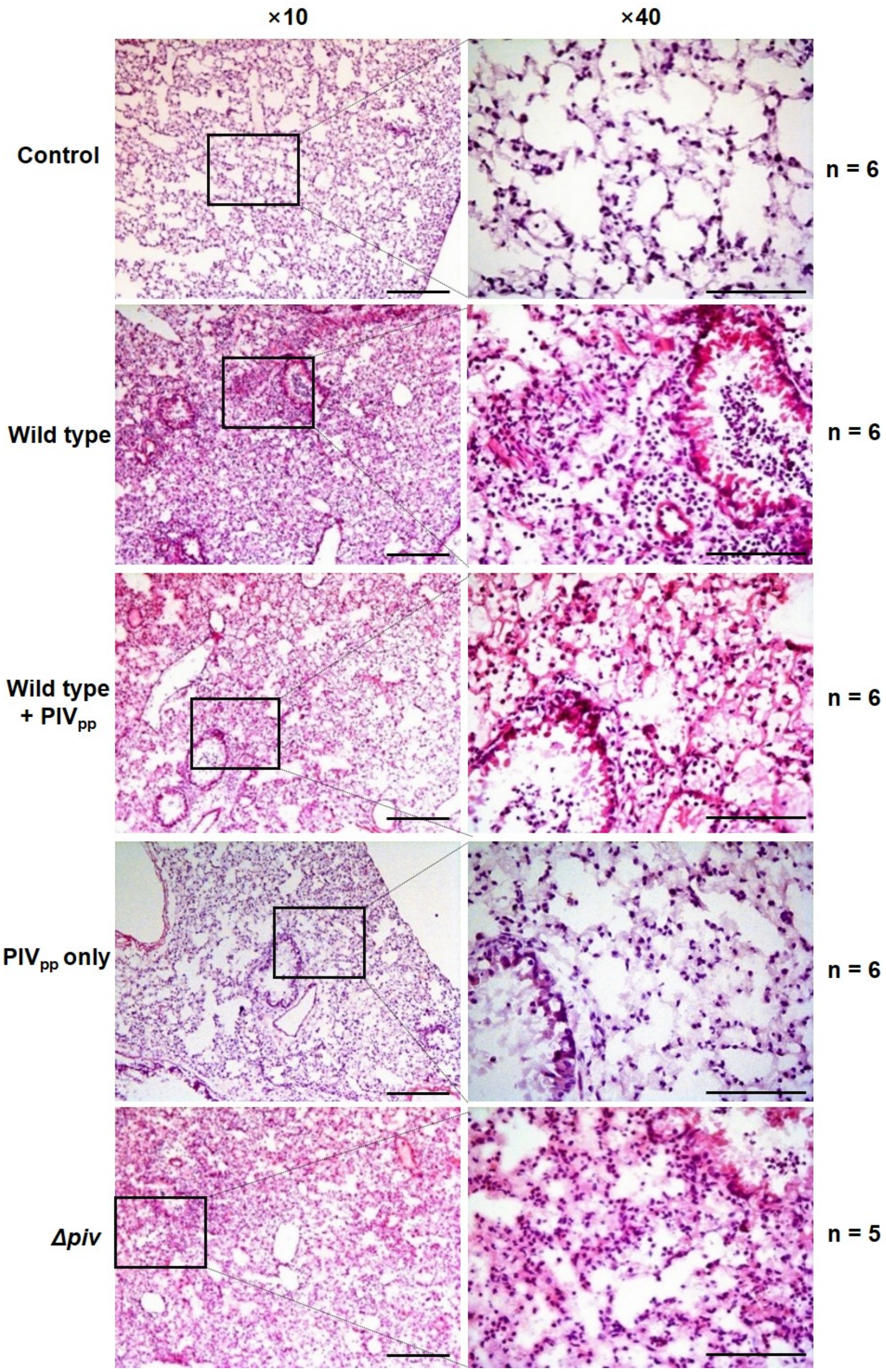

×10
×40
Control
n = 6
Wild type
n = 6
Wild type
+ PIV$_{pp}$
n = 6
PIV$_{pp}$ only
n = 6
Δpiv
n = 5

Fig. S4. - continued

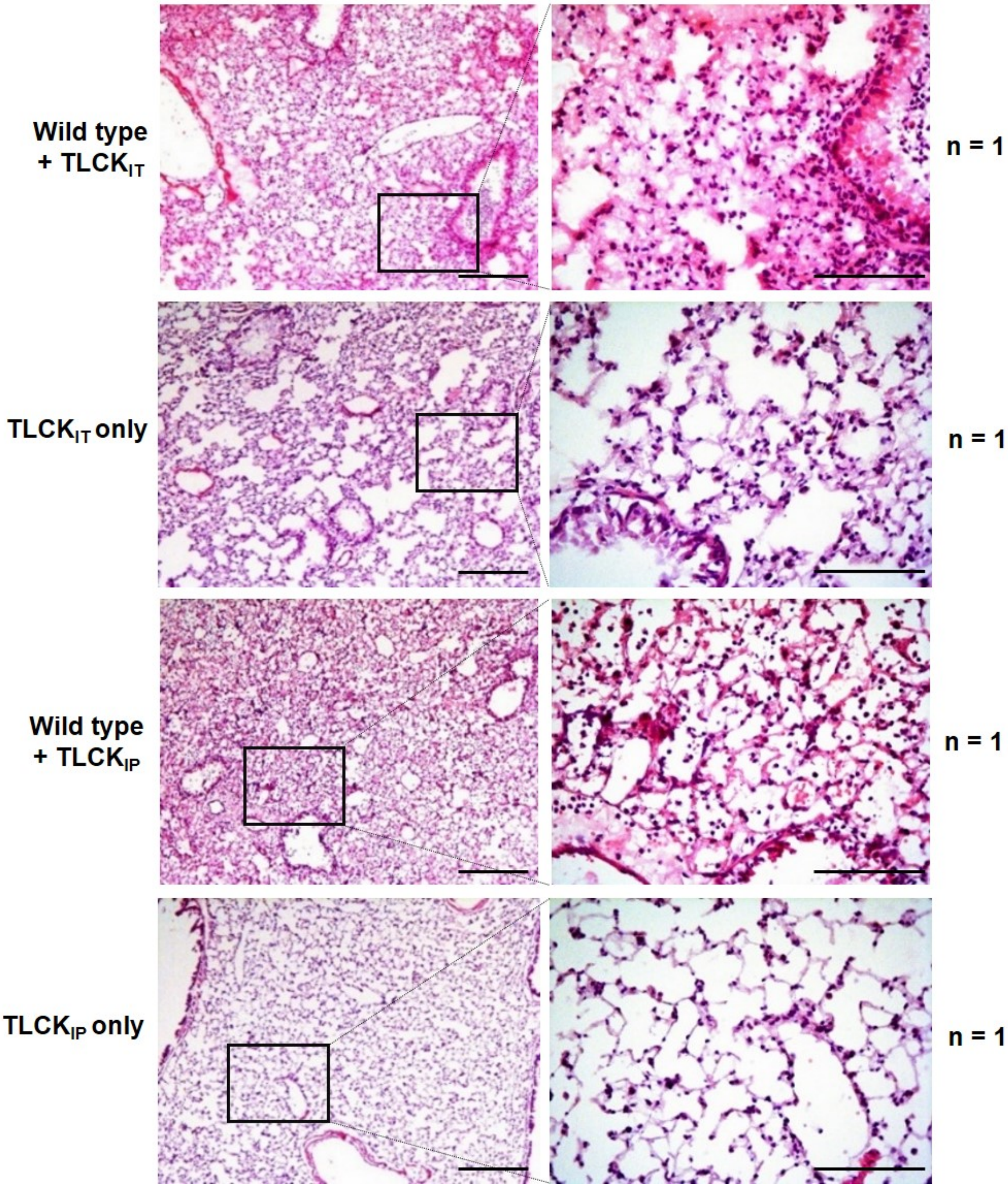

Fig. S4. 3rd trial of the acute lung infection with TLCK-treated *P. aeruginosa*. The same experiment in Fig 4 was additionally repeated and the TLCK treatment groups were added. The number of mice used in each infection were indicated (n). TLCK$_{IT}$ indicates the direct treatment of *P. aeruginosa* with 1 mM TLCK and TLCK$_{IP}$ indicates the intraperitoneal injection of TLCK to mice at 10 mg/weight (kg), separately from the *P. aeruginosa* inoculation.

Reference

1. Pearson JP, Pesci EC, Iglewski BH. 1997. Roles of Pseudomonas aeruginosa las and rhl quorum-sensing systems in control of elastase and rhamnolipid biosynthesis genes. Journal of Bacteriology 179:5756-67.
2. Park SJ, Kim SK, So YI, Park HY, Li XH, Yeom DH, Lee MN, Lee BL, Lee JH. 2014. Protease IV, a quorum sensing-dependent protease of Pseudomonas aeruginosa modulates insect innate immunity. Mol Microbiol 94:1298-314.
3. Lee MN, Kim SK, Li XH, Lee JH. 2014. Bacterial virulence analysis using brine shrimp as an infection model in relation to the importance of quorum sensing and proteases. Journal of General and Applied Microbiology 60:169-74.
4. Kim SK, Park SJ, Li XH, Choi YS, Im DS, Lee JH. 2018. Bacterial ornithine lipid, a surrogate membrane lipid under phosphate-limiting conditions, plays important roles in bacterial persistence and interaction with host. Environ Microbiol 20:3992-4008.
5. Oh J, Li XH, Kim SK, Lee JH. 2017. Post-secretional activation of Protease IV by quorum sensing in Pseudomonas aeruginosa. Sci Rep 7:4416.
6. Yang Y, Wang J, Han T, Liu T, Wang C, Xiao J, Mu C, Li R, Yu F, Shi H. 2015. Ovarian Transcriptome Analysis of Portunus trituberculatus Provides Insights into Genes Expressed during Phase III and IV Development. PLoS One 10:e0138862.
7. Overbergh L, Valckx D, Waer M, Mathieu C. 1999. Quantification of murine cytokine mRNAs using real time quantitative Reverse Transcriptase PCR. Cytokine 11:305-12.
8. Schmidt RL, Lenz LL. 2012. Distinct licensing of IL-18 and IL-1beta secretion in response to NLRP3 inflammasome activation. PLoS One 7:e45186.
